# Optimization of intra‐voxel incoherent motion measurement in diffusion‐weighted imaging of breast cancer

**DOI:** 10.1002/acm2.12065

**Published:** 2017-03-27

**Authors:** Wenjing Chen, Juan Zhang, Dan Long, Zhenchang Wang, Jian‐Ming Zhu

**Affiliations:** ^1^ Institute for Biomedical Engineering China Jiliang University Hangzhou Zhejiang China; ^2^ Department of Radiology Zhejiang Cancer Hospital Hangzhou Zhejiang China; ^3^ Department of Radiology and Center for Medical Imaging Research Beijing Friendship Hospital Capital Medical University Beijing China

**Keywords:** breast cancer, b‐value, diffusion‐weighted imaging, intra‐voxel incoherent motion

## Abstract

**Purpose:**

The purpose of this study was to optimize intra‐voxel incoherent motion (IVIM) measurement in diffusion‐weighted imaging (DWI) of breast cancer by separating perfusion and diffusion effects through the determination of an optimal threshold b‐value, thus benign and cancerous breast tissues can be accurately differentiated using IVIM‐derived diffusion and perfusion parameters.

**Materials and Methods:**

Twenty‐eight patients, with biopsy‐confirmed breast cancers, were studied with a 3T MRI scanner, using T1‐weighted dynamic contrast‐enhanced MRI images, and diffusion‐weighted images with nine b‐values, ranging from 0 to 1000 s/mm². IVIM‐derived parameter maps for tissue diffusion coefficients *D*, perfusion fraction *f*, and pseudo‐diffusion coefficients D* were computed using the segmented fitting method with optimized threshold b‐value, and the sum of squared residuals (SSR) were calculated for IVIM‐derived parameters in different breast lesions.

**Results:**

The IVIM analysis method developed in this work can separate perfusion and diffusion effects with the optimal threshold b‐value of 300 s/mm², and the results of diffusion and perfusion parameters from IVIM analysis can be used to differentiate pathological changes in breast tissues. It was found that the averages and standard deviations of the diffusion and perfusion parameters, *D*,* f*, D*, are the following, for malignant, benign and normal breast tissues respectively: *D* (0.813 ± 0.225 × 10^−3^ mm^2^/s, 1.437 ± 0.538 × 10^−3^ mm^2^/s, 1.838 ± 0.213 × 10^−3^ mm^2^/s), *f* (10.73 ± 3.44%, 7.86 ± 3.70%, 8.92 ± 3.72%), D* (15.23 ± 12.17×10^−3^ mm²/s, 12.02 ± 3.19 × 10^−3^ mm^2^/s, 12.03 ± 7.21 × 10^−3^ mm^2^/s).

**Conclusion:**

IVIM‐derived diffusion and perfusion parameter maps depend highly on the choice of threshold b‐value. Using the methodology developed in this work, and with the optimized threshold b‐value, the diffusion and perfusion parameters of breast tissues can be accurately assessed, making IVIM MRI a technique of choice for differential diagnosis of breast cancer.

## 1 Introduction

Breast cancer is one of the most common malignant tumors in females, and is the second leading cause of cancer death in women.^1^ Magnetic resonance imaging (MRI) has been used more and more widely for the detection and diagnosis of breast cancers.^2^ As a functional MRI technique, diffusion, and perfusion imaging are two of the most popular methods in breast cancer imaging.^3^ Diffusion imaging or diffusion‐weighted MRI (DWI) utilizes the Brownian motion effects of water molecules in the tissue intra‐ and extracellular spaces, has the potential to provide biological information on tumor blood micro‐vasculature at the cellular levels.^4^, ^5^ Conventional DWI uses diffusion‐weighting factors, the so called b‐values, to derive the diffusion parameters. Based on MRI water signal attenuation model, the apparent diffusion coefficients (ADC) can be computed and images of diffusion parameters such as ADC maps can be reconstructed.^6^ Due to active tumor cell growth pattern, diffusion of water molecules in malignant tissues is usually restricted by more tightened cellular membrane microstructure, and the ADC values in tumors thus are reduced. However in DWI images, malignant tissues show higher signal intensities. DWI therefore can be used to detect, monitor, and predict the tumor growth.^2^, ^3^

DWI measurement may be affected by contributions from perfusion phenomena.^7^, ^8^, ^9^ Due to random distribution of capillary network in tissue, at the single voxel level, measured ADC values are typically higher than actual values, because of the contribution from blood flow perfusion effects from intra‐voxel incoherent motion. DWI measurement thus reflect contribution also from tissue perfusion effects, as the microscopic blood flow in a randomly oriented capillary network produce a pseudo‐diffusion contribution to the overall diffusion‐weighted (DW) MR signal.

Le Bihan et al.^7^, ^8^ demonstrated that blood microcirculation in capillary network (perfusion) was able to change DW signal intensities at low b‐values, and the intra‐voxel incoherent motion (IVIM) theory was proposed to account for the molecular diffusion contribution driven by thermal energy as well as perfusion‐based pseudo‐diffusion contribution. In IVIM theory, diffusion measurement contribution has two parts: true diffusion part and pseudo‐diffusion part from perfusion. The selection of b‐values in DWI measurement was considered to have strong effects for IVIM analysis and its derived diffusion parameters.^10^

In recent years, IVIM measurement in the imaging of different organs has gained more attention, for example, in normal livers and liver cirrhosis,^11^, ^12^, ^13^, ^14^ in kidney,^15^, ^16^, ^17^ and in the prostate.^10^, ^18^ However, different range of b‐values were used in the clinical measurement of IVIM parameters from different institutions,^2^, ^19^, ^20^ and there were no agreed method and optimal range of b‐values to separate the diffusion and perfusion effects. In clinical application, most studies suggested that perfusion may have more dominating effects when b‐values are less than 200 s/mm^2^, and selection of different b‐value thresholds will result in different IVIM parameters.^21^, ^22^, ^23^ Wurnig et al. proposed a computation method to evaluate IVIM parameters by optimal selection of b‐value thresholds for the separation of perfusion and diffusion effects.^24^

In diffusion‐weighted imaging studies of breast tissue with multiple b‐values, the benefit of using IVIM MRI is that it can result in information of tissue perfusion without the use of the “traditional” intravenously injected MR contrast agents, in additional to the diffusion parameters. IVIM analysis can extract detailed information about tissue diffusion and perfusion simultaneously, and has the potential to evaluate tissue perfusion noninvasively. There are previous studies indicating the clinical diagnosis potential of IVIM MRI for breast cancers.^21^, ^22^, ^23^, ^25^, ^26^ However, currently published studies used different parameters and methods for IVIM analysis, limited data showed that the IVIM‐derived parameters were very different with large errors, especially for parameters *f* and D*.^22^ The purpose of this study is therefore to optimize IVIM measurement and analysis for breast cancer patients, specifically, an optimized threshold b‐value will be sought out so that diffusion and perfusion effects in three types of breast tissues and lesions can be separated.

## 2 Materials and methods

### 2.A Study population

This study was approved by our institutions’ review boards (IRB), consents to participate in the study were obtained from each patient before MRI examination were performed. In total, 28 women who were diagnosed with breast tumors were recruited for this study, among them, 18 tumor lesions were diagnosed as malignant (invasive ductal carcinoma, IDC), and 11 tumors were benign lesions (one of them has two benign lesions) . The mean age of the patients was 47 years old, ranging from 15 to 62 years old.

### 2.B MRI image acquisition

All patients’ MRI studies were conducted using a clinical 3T MRI system (Magnetom Verio, Siemens, Erlangen, Germany). Patients were lying down in their head‐first prone positions with their bilateral breasts naturally hung in the middle of a 16‐channel bilateral SENSE breast coil. All patients were scanned with T1‐weighted contrast‐enhanced MRI and diffusion‐weighted MRI sequence with multiple b‐values. In additional to 3‐plane localizer, axial T1‐weighted (repetition time/echo time (TR/TE = 700/10 ms), field of view (FOV) 320 × 320 mm^2^, matrix 640 × 640, 24 slices) and T2‐weighted images (TR/TE = 5800/84 ms, FOV 300 × 300 mm^2^, matrix 640 × 640, 24 slices) with and without fat suppression were acquired. For dynamic contrast‐enhanced MRI, 25 ml of Gd‐DTPA was delivered via intravenous power injector (Medrad, Pittsburgh, PA USA), followed by 25 ml of saline solution at the delivery rate of 2 ml/s. The detailed acquisition parameters were as the following: TR/TE = 4.5/1.6 ms, FOV 340 × 340 mm^2^, matrix 896 × 896, 112 total number of slices. Total scan time was 4 minutes 57 s. DWI images used for IVIM measurements were acquired with an EPI‐based DWI sequence, and in total, nine b‐values (0, 50, 100, 150, 200, 300, 400, 800, 1000 s/mm^2^) were applied in the IVIM image acquisition. DWI acquisition was done before the dynamic contrast‐enhanced pulse sequence. For each b‐value DWI measurement, all three orthogonal x, y, z gradients were used in three acquisitions. All DWI images were acquired at axial planes, and image acquisition parameters were the following: TE, 67 ms; TR, 6600 ms; flip angle, 90°; image matrix size, 120 × 224; FOV, 187 × 350 mm^2^; slice thickness, 5 mm; slice gap, 6.5 mm, number of averages, 2, number of slices 18. Total scan time for IVIM measurement was 5 minutes and 50 seconds.

### 2.C IVIM analysis

Parametric maps of diffusion and perfusion with IVIM image analysis were all reconstructed with MATLAB program (Mathworks, Natick, MA, USA). In IVIM model, the signal intensity curves from multiple b‐value DWI experiments were expressed with the following formula:

(1) $$S\left(b\right)/S_{0}=f\;\exp\left(-bD^{*}\right)+\left(1-f\right)\exp\left(-bD\right)$$

Where *S*(*b*) and *S*_0_ denote the diffusion‐weighted signal intensities of the pixels with and without diffusion‐encoding gradients (indicated by the b‐value), respectively. *D* is the apparent diffusion coefficient as reflected by pure molecular diffusion. *f* denotes the perfusion fraction. D* denotes the pseudo‐diffusion coefficient.

The computation of *D* used the “traditional” mono‐exponential diffusion model from diffusion‐weighted images at multiple b‐values, with the use of Eq. (2):

(2) $$S\left(b\right)/S_{0}=\exp\left(-bD\right)$$

To separate diffusion and perfusion in the presence of IVIM effects, a segmented bi‐exponential analysis method was used.^21^, ^22^, ^23^ Since perfusion contribution is negligible in high b‐values DWI measurements, *D* maps were first computed with the polynomial fitting method using Eq. (2) from the DWI images acquired with higher b‐values, where the lowest b‐value among the higher b‐values DWI for *D* map computation is named as threshold b‐value.

Secondly, the perfusion fraction (*f*) is calculated according to Eq. (3),

(3) $$f=\left(S_{0}-S_{int}\right)/S_{0}$$

Where *S*_int_ denotes the intercept pixel signal intensity when b‐value extrapolates to 0 from the fitting curves. To calculate the pseudo‐diffusion coefficient (*D* ^*^), a bi‐exponential model of diffusion, as shown in Eq. (1), was used, which was originally described by Le Bihan et al.^8^ D* is calculated using nonlinear least square fitting algorithm by selecting all the b‐values. The trust‐region‐reflective algorithm^27^ was used in this step.

The optimal threshold b‐values were derived from the following three steps: in the first step, *D‐*50 was derived from the first‐order poly‐nominal fitting using Eq. (2) with all b‐values between 50–1000 s/mm^2^. Repeating the above steps, *D*‐100, *D*‐150, *D*‐200, *D*‐300, *D*‐400, *D*‐800 were derived with b‐values between 100–1000 s/mm^2^ till to 800–1000 s/mm^2^, here, *D*‐100, for example, denotes the threshold b‐value equals 100 s/mm^2^. In the second step, seven *f* values were calculated using Eq. (3) based on different *D*‐b_value_. In the third step, using the *D*‐b_value_ and *f*‐b_value_ calculated from above two steps, seven D*‐b_value_ were calculated using Eq. (1). Finally, using Eq. (4), the sum of square residuals (SSR) were calculated to evaluate the difference between the measured data *y*_*i*_ and fitted results *f*(*x*_*i*_), so that the threshold b‐values can be determined where the effect of diffusion and perfusion is separated. *n* denoted the number of measured data.

(4) $$SSR=\sum_{i=1}^{n}{\left(y_{i}-f\left(x_{i}\right)\right)}^{2}$$

### 2.D Region of interest selection

Lesions were identified through a combination of MRI T1/T2/DCE images and pathology biopsy examination results. The regions of interest (ROI) from the lesions were selected in the slices that contain the maximum lesion areas**.** For IVIM analysis, ADC maps (*D* maps) were calculated using Eq. (2) from the DWI image acquired with the minimal b = 50 s/mm^2^, an ROI was then drawn on the *D*‐50 map with free‐hand, and the same ROI was copied to the *f* and D* maps in MATLAB software. In addition, the sum of squared residuals (SSR) was calculated for the same ROIs. This ensure that the three IVIM‐derived parameters were computed and evaluated from the identical ROIs, thus to maintain the accuracy and precision of the IVIM analysis. The selection of ROIs was drawn together by two radiologists, both of them have over 10 years of experience in breast MRI.

## 3 Results

The final study included a total of 28 patients, among them, 18 tumor lesions were diagnosed as malignant (invasive ductal carcinoma, IDC), and 11 tumors were classified as benign lesions (one of them has two benign lesions). Hence, a total of 29 breast lesions were included in the final analysis and were assessed with DWI and the gadolinium‐enhanced MRI examination. Lesion identification and classification were confirmed through a combination of pathology biopsy examination results and MRI T1/T2/DWI/DCE images. The regions of interest (ROI) from the lesions were selected in the slices that contain the maximum lesion areas**.**

**Figure 1:**
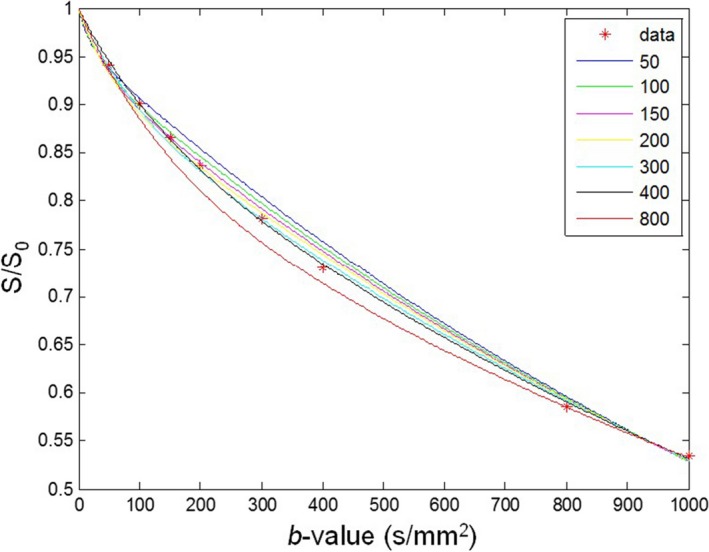
Fitting curves obtained with different threshold b‐values from an ROI within a malignant lesion of a patient. Red dots denote averaged signal intensities measured from the ROI at different threshold b‐values. The numbers on the top right side of the figure represent each threshold b‐values.

Fitting results obtained with different threshold b‐values from an ROI within a malignant lesion of a patient were shown in Fig. 1. In this figure, diffusion‐weighted signal decays were plotted against threshold b‐values. Seven fitting curves were generated for seven threshold b‐values. The fitting curves separate with measured data when the threshold b‐values were 50, 100, and 800 s/mm^2^, and the fitting results are not as close as those at the threshold b‐values equal to 150, 200, 300, and 400 s/mm^2^.

**Figure 2:**
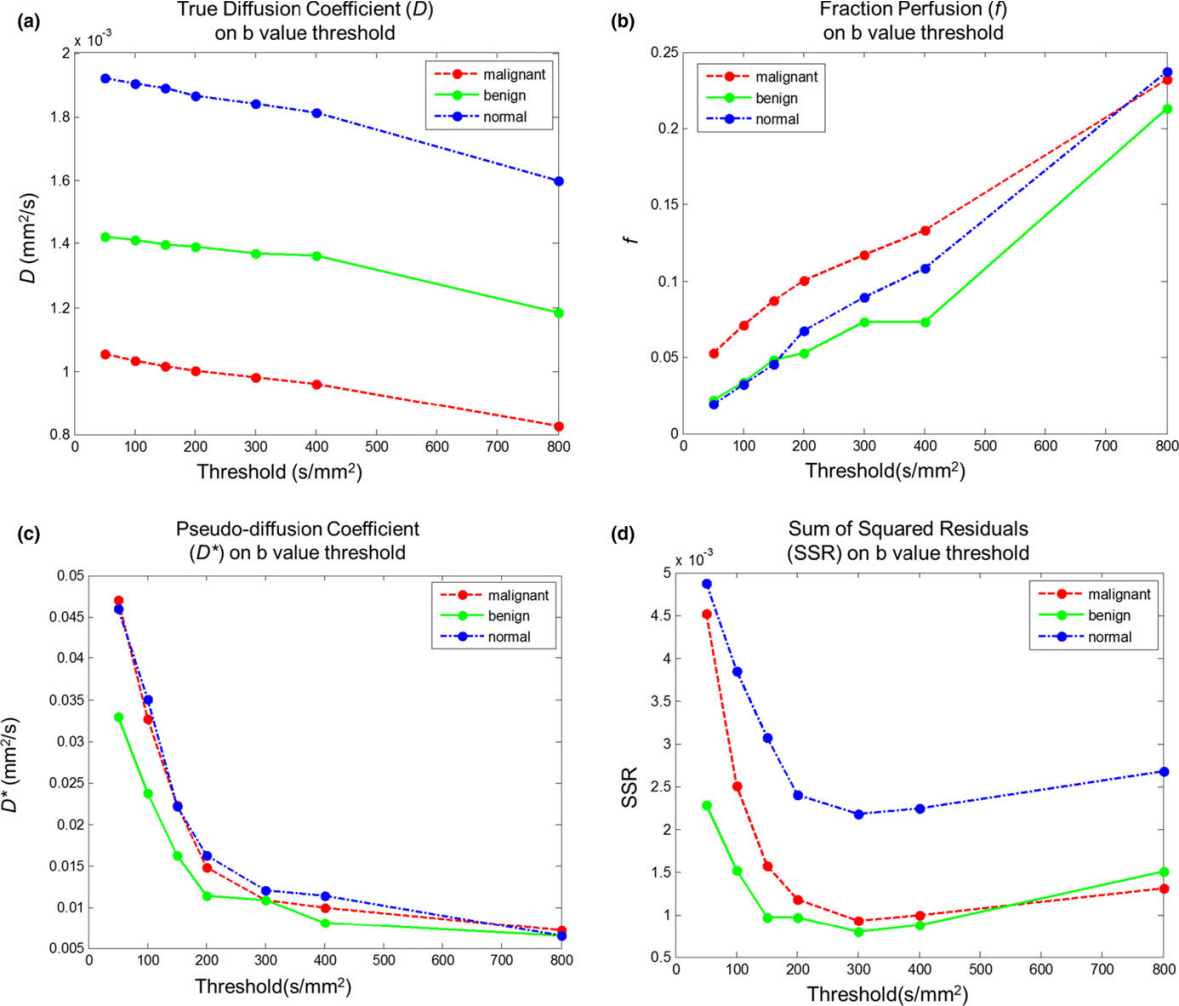
Computation results for three parameters *D* (a), *f* (b), and D* (c), and SSR (d), under different threshold b‐values from three different ROIs, each containing with a malignant lesion, a benign lesion and a normal tissue. *X*‐axis is the threshold b‐value.

Calculated results for three IVIM parameters *D*,* f*, D*, and SSR, under different threshold b‐values with three different ROIs, each contained with a malignant lesion ROI, benign lesion ROI and a normal tissue ROI, are shown in Fig. 2. The average dimensions of the ROI for malignant, benign, and normal fibroglandular tissue were 102 mm^2^, 78.37 mm^2^, and 36.28 mm^2^, respectively. As the threshold b‐values increase, the diffusion coefficient *D* of tissue tends to decrease; also, the *D* values for malignant lesions, benign lesions, and normal tissues decrease to different extends. This shows that the *D* values could potentially provide some preliminary differential classification of breast tissue's tumor invasiveness. The perfusion fraction of different tissues increases with threshold b‐values. When the threshold b‐value approaches to 0, the pseudo‐diffusion coefficient becomes maximum, indicating dominating effects from tissue perfusion. When the threshold b‐value increases, D* deceases, the sum of squared residuals SSR decrease initially and then increase.

**Figure 3:**
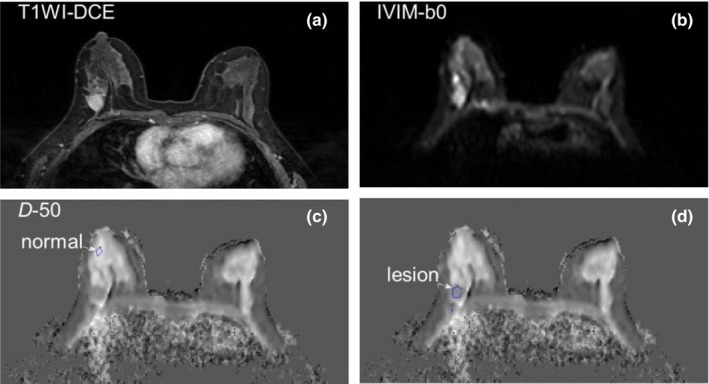
Selection of ROIs for IVIM analysis from a slice in a patient case study. (a) T1‐weighted contrast‐enhanced breast image; (b) T2‐weight image of the same slice; (c) and (d) *D* maps calculated with threshold b‐value at 50 s/mm^2^, (c) is labeled with selected ROI location for normal tissue, and (d) is labeled with selected ROI location for a malignant lesion.

The selection of ROI for IVIM analysis from a slice in a patient case study is shown in Fig. 3. Figure 3(a) shows an example of T1‐weighted dynamic contrast‐enhanced breast images; Fig. 3(b) shows a T2‐weight image of the same slice; Figs. 3(c) and 3(d) represent *D* maps calculated with threshold b‐value at 50 s/mm^2^, where Fig. 3(c) is labeled with selected ROI location for normal tissue, and Fig. 3(d) is labeled with selected ROI location for a malignant lesion.

**Figure 4:**
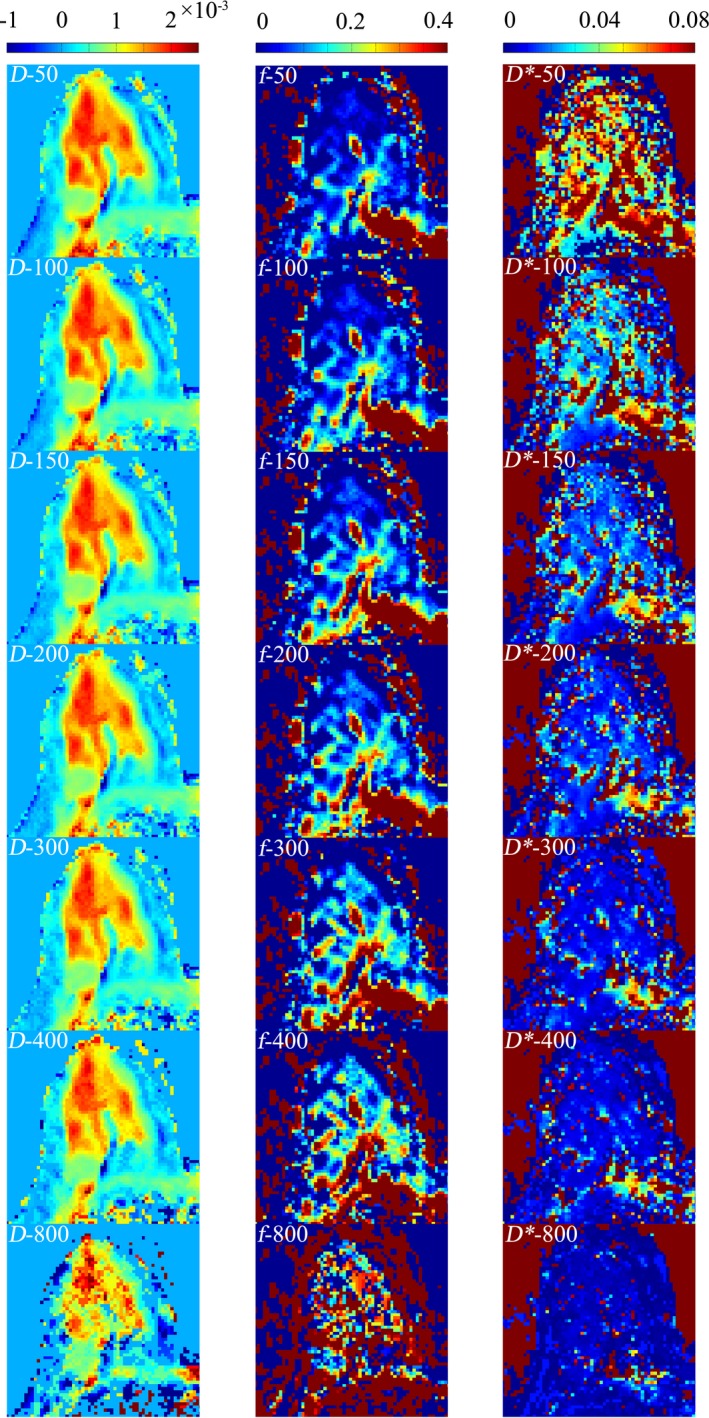
Three sets of IVIM‐derived parameter maps for *D* (mm^2^/s), *f*, and D* (mm^2^/s), from one of the patients using different threshold b‐values.

Figure 4 showed three series of representative IVIM‐derived parameter maps for *D*,* f*, and D*, from a slice of a patient case study using different threshold b‐values for computation. The slice was chosen the one for all the displayed maps.

The results of the mean value and standard deviation for three IVIM parameters *D*,* f*, and D*, from all patients’ ROIs containing with malignant and benign lesions, as well as normal tissues, using seven threshold b‐values for computation, are listed in Table 1. For normal breast tissue, benign breast lesions, and malignant lesions, the *D* value can be ranged from 1.597 to 1.919 × 10^−3^ mm^2^/s, 1.304 to 1.492 × 10^−3^ mm^2^/s, and 0.708 to 0.880 × 10^−3^ mm^2^/s respectively; *f* can be ranged extensively from 1.93% to 23.7%, 2.86% to 17.95%, and 4.47% to 18.17% respectively for three types of tissue. Finally, D* ranged from 6.49 to 45.95 × 10^−3^ mm^2^/s, 8.96 to 44.21 × 10^−3^ mm^2^/s, and 9.93 to 47.32 × 10^−3^ mm^2^/s, respectively, for three types of tissues. From these results, the choice of threshold b‐values has huge impact on *f*, and D* values in IVIM analysis.

**Table 1:** Results of the mean and standard deviation of three IVIM‐derived parameters *D* (A), *f* (B), and D*(C), from all the patients’ ROIs containing with malignant and benign lesions, as well as normal tissues

Threshold (s/mm^2^)	Normal	Benign	Malignant
A	*D*(×10^−3^ mm^2^/s)		
50	1.919 ± 0.203	1.492 ± 0.533	0.880 ± 0.251
100	1.904 ± 0.205	1.480 ± 0.534	0.865 ± 0.245
150	1.888 ± 0.207	1.469 ± 0.536	0.853 ± 0.238
200	1.864 ± 0.211	1.455 ± 0.537	0.832 ± 0.277
300	1.838 ± 0.213	1.437 ± 0.538	0.813 ± 0.225
400	1.810 ± 0.218	1.420 ± 0.536	0.811 ± 0.226
800	1.597 ± 0.328	1.304 ± 0.545	0.708 ± 0.224
B	*f* (%)		
50	1.93 ± 1.76	2.86 ± 2.28	4.47 ± 2.23
100	3.21 ± 1.86	4.02 ± 2.73	5.85 ± 2.25
150	4.55 ± 2.50	5.03 ± 3.15	6.89 ± 2.22
200	6.79 ± 3.36	6.14 ± 3.49	8.91 ± 2.90
300	8.92 ± 3.72	7.86 ± 3.70	10.73 ± 3.44
400	10.86 ± 4.70	8.91 ± 4.02	10.59 ± 3.50
800	23.70 ± 11.17	17.95 ± 7.18	18.17 ± 9.64
C	D*(×10^−3^ mm^2^/s)		
50	45.95 ± 12.66	44.21 ± 7.05	47.32 ± 14.31
100	35.08 ± 15.37	29.26 ± 6.93	36.43 ± 15.50
150	22.22 ± 9.81	23.52 ± 8.31	28.53 ± 17.07
200	16.24 ± 10.71	17.27 ± 6.40	21.95 ± 15.94
300	12.03 ± 7.21	12.02 ± 3.19	15.23 ± 12.17
400	11.34 ± 7.80	9.39 ± 2.17	18.52 ± 13.92
800	6.49 ± 7.03	8.96 ± 4.58	9.93 ± 5.36

To determine the optimal threshold b‐value for IVIM analysis of breast tissue at 3T, minimum SSR for each patient was used to extract the optimal threshold, the results are shown in Table 2, with the total number of patients for ROIs from malignant and benign lesions with the specific threshold b‐values. The result from this study showed that the optimal threshold b‐value to separate diffusion and perfusion effects for accurate IVIM analysis is 300 s/mm^2^. This study also determined that the averages and standard deviations of the diffusion and perfusion parameters, *D*,* f*, and D*, from this IVIM study, are the following, for malignant, benign, and normal breast tissues respectively: *D* (0.813 ± 0.225 × 10^−3^ mm^2^/s, 1.437 ± 0.538 × 10^−3^ mm^2^/s, 1.838 ± 0.213 × 10^−3^ mm^2^/s), *f* (10.73 ± 3.44%, 7.86 ± 3.70%, 8.92 ± 3.72%), D* (15.23 ± 12.17 × 10^−3^ mm^2^/s, 12.02 ± 3.19 × 10^−3^ mm^2^/s, 12.03 ± 7.21 × 10^−3^ mm^2^/s).

**Table 2:** Numbers of patient cases that yield the optimal threshold *b*‐value with the smallest residuals for malignant and benign tissues from all patients; there is a maximum number of patients (9 + 7, 16) whose IVIM analysis results indicated the optimal threshold b‐value is 300

Threshold b‐value (s/mm^2^)	200	300	400	Median
Malignant	4	9	5	300
Benign		7	4	300

## 4 Discussion

Intra‐voxel incoherent motion (IVIM) diffusion‐weighted imaging has recently gained an increasingly interest due to its potential to insight tissue microenvironment with both tissue diffusion and perfusion information. Several studies^23^, ^25^, ^26^, ^28^, ^29^ have shown that using different threshold b‐values for IVIM analysis would lead to very different results of perfusion and diffusion parameters.^23^, ^25^, ^26^, ^28^, ^29^ As threshold b‐value increases, measured *D* value tends to decrease, while measured *f* and D* values have different effects. Studies from Bokacheva et al.,^25^ Liu et al.,^23^ and Borlinhas et al.^26^ showed measured ADC values were larger than *D* values, this indicated the micro‐vasculature effects to the diffusion coefficients. In the IVIM analysis of breast tissues, several analytical methods have been reported, including direct estimation of IVIM parameters with a nonlinear‐fitting algorithm, a segmented analysis procedure. Also, threshold b‐values ranging from 100 to 400 s/mm^2^ have been used in several studies,^23^, ^25^, ^26^, ^28^, ^29^ and no conclusion has been made on the effects of threshold b‐value selection with regards to the results of IVIM‐derived parameters. The purpose of this study was to optimize intra‐voxel incoherent motion (IVIM) based diffusion‐weighted imaging (DWI) of breast cancer by separating perfusion and diffusion effects so that reliable computation of diffusion and perfusion‐related parameters from IVIM DWI signals can be obtained. This was specifically achieved through the determination of an optimal threshold b‐value, so that benign and cancerous breast tissues can be accurately differentiated using IVIM‐derived diffusion and perfusion parameters.

As with other recent studies,^30^, ^31^, ^32^ segmented fitting method has been used in the present work to derive the parameters through multiple fitting steps. Segmented fitting method is known to prevent over‐fitting, and computation errors can be reduced.^33^, ^34^, ^35^ There are typically two methods of segmented fitting^11^, ^12^, ^13^, ^18^, ^21^, ^23^: two‐step fitting and three‐step fitting, and the most important step is the calculation of *D* maps with higher b‐value images using Eq. (2). A two‐step segmented fitting method uses high b‐value DWIs to calculate the *D* maps with a simplified linear‐fitting Equation [see Eq. (2)], then *f* and D* are calculated using a nonlinear‐fitting algorithm for all b‐value DWIs.^23^ In general, due to limited data sampling and small perfusion fraction, two‐step segmented fitting process ill‐conditioned, thus may produce large errors. A three‐step segmented fitting method is used in this work due to its fitting robustness and computation stability.

The determination of optimal threshold b‐value is the most important step in segmented fitting procedure. If no optimal threshold b‐value is chosen for the first step of *D* map computation, the estimation of IVIM perfusion fraction and pseudo‐diffusion parameters will generate large errors, since diffusion and perfusion effects are not well separated in the IVIM analysis.

In some recent studies with breast cancer patients,^28^ a minimal b‐value of 120–400 s/mm^2^ has been used in their studies. A study by Bokacheva et al.,^25^ used a b‐value of 120 s/mm² as the threshold, and the three‐step segmented fitting method was used for IVIM analysis. Another study by Cho et al.,^28^ using b‐value of 150 s/mm² as the threshold, compared four analysis methods, free fitting, segmented fitting, conventional, and optimized b‐value selection method, and found that segmented fitting method combined with optimized b‐value selection is the optimal method. Other studies have used their optimal threshold b‐value of 200 s/mm^2^ from their liver studies as the threshold b‐value for breast clinical diagnosis studies.^14^, ^36^ In other disease studies, Chandarana et al.^16^ used a b‐value of 250 s/mm^2^ as the threshold, and Koh et al.^2^ used b‐value of 100 s/mm^2^ as the threshold for their liver studies. All these studies indicated the importance of threshold b‐value selection for segmented fitting method in IVIM analysis.

In this current study, we have determined that the optimal threshold b‐value to separate diffusion and perfusion effects for accurate IVIM analysis is 300 s/mm^2^, as showed in the Results section. Our selection came from the detailed three‐step analysis by comparing seven groups of IVIM‐derived parameters, and nonlinear curve fitting was performed with experimental data (Fig. 1). Through quantitative SSR analysis (Fig. 2(d)) for all three tissue types, it was found that the optimal threshold b‐value for malignant and benign lesions were 300 s/mm^2^, while normal breast tissue was 400 s/mm^2^. For differential diagnosis, and in comparing with our experimental results, it was determined that the optimal threshold b‐value of IVIM breast tissue imaging and analysis is 300 s/mm^2^ (Table 2).

Finally, the following IVIM parameters were obtained in this study for malignant, benign and normal breast tissues, respectively: *D* (0.813 ± 0.225 × 10^−3^ mm^2^/s, 1.437 ± 0.538 × 10^−3^ mm^2^/s, 1.838 ± 0.213 × 10^−3^ mm^2^/s), *f* (10.73 ± 3.44%, 7.86 ± 3.70%, 8.92 ± 3.72%), D* (15.23 ± 12.17 × 10^−3^ mm^2^/s, 12.02 ± 3.19 × 10^−3^ mm^2^/s, 12.03 ± 7.21 × 10^−3^ mm^2^/s). Comparing to the study results by Bokacheva et al.,^25^ for malignant breast lesion: *D* (1.29 ± 0.28 × 10^−3^ mm^2^/s), *f* (6.4 ± 3.1%), D* (21.7 ± 11.0 × 10^−3^ mm^2^/s); and benign breast lesion: *D* (1.56 ± 0.28 × 10^−3^ mm^2^/s), *f* (3.1 ± 3.3%), D* (27.6 ± 34 × 10^−3^ mm^2^/s); and the study results by Cho et al.*,* 28 for malignant breast tissue was: *D* (1.195 ± 0.471 × 10^−3^ mm^2^/s), *f* (16.83 ± 9.06 %), and D* (13.183 ± 6.529 × 10^−3^ mm^2^/s), our ADC result for malignant breast lesions (*D =* 0.813 ± 0.225 × 10^−3^ mm^2^/s) are relatively lower than the other two studies (*D* = 1.29 ± 0.28 × 10^−3^ mm^2^/s, and 1.195 ± 0.471 × 10^−3^ mm^2^/s). This might be due to the fact that much lower threshold b‐values were used in their studies, as indicated earlier. When much lower threshold b‐values are used in IVIM analysis, the perfusion related contribution, or pseudo‐diffusion contribution, to the diffusion ADC measurement is much more involved, therefore, leading to higher *D* measurement results. This supported our result for optimization of threshold b‐value, and also indicated that, with the current IVIM analysis method, the effects of perfusion and diffusion are clearly separated.

The perfusion fraction (*f* = 10.73 ± 3.44%) result for malignant breast lesions fitted in the middle of the other two studies (*f* = 6.4 ± 3.1% and 16.83 ± 9.06%). The pseudo‐perfusion coefficient (D* = 15.23 ± 12.17 ×10^−3^ mm^2^/s) result for malignant breast lesions was also in the middle of the other two studies (21.7 ± 11.0 × 10^−3^ mm^2^/s and 13.183 ± 6.529 × 10^−3^ mm^2^/s, respectively). This indicated our method of IVIM analysis is robust and accurate in comparison with other recently published methods in literature.

There are some limitations in this study. Similar to many other studies of IVIM DWI, our study was limited by the total number of b‐value DWI measurements, for example, we used only four low b‐value DWI image sets, which are typically for b = 50, 100, 150, 200 s/mm^2^ DWIs, and it is considered relatively small number of b‐values DWI data for segmented fitting method of IVIM analysis. Lemke et al. indicated in their study that the most suitable number of b‐value DWIs for their method of IVIM analysis is 10.^19^ While increasing the total number of b‐value DWIs may improve the robustness and fitting accuracy of our IVIM analysis, the variance among the scans for different b‐value DWIs with effects such as motion or other artifacts would result in a much less accurate fitting, leading to inaccurate computation of IVIM parameters. If the total number of b‐value DWI is increased, the IVIM‐derived parameter maps could be potentially more accurate. For some patients, the segmented fitting method sometime would fail to generate the high quality IVIM parameter maps because the segmented fitting needed a minimal number of data points below and above the threshold b‐values, to generate high quality IVIM parameters.

In conclusion, IVIM‐derived diffusion and perfusion parameter maps depend highly on the choice of threshold b‐value. Using the methodology developed in this work, and with the optimized threshold b‐value, the diffusion and perfusion parameters of breast tissues can be accurately assessed, making IVIM MRI a technique of choice for differential diagnosis of breast cancer. While comprehensive IVIM studies and comparison of all breast tissues for IVIM‐derived parameters entitled further studies, our preliminary results in this current study validated our methodology for further IVIM studies in more patient studies and can be applied in other organ or disease sites as well.

## Acknowledgment

A Distinguished Visiting Professorship (JMZ) from the Provincial Government of Zhejiang, China is greatly appreciated. This work was partially funded by Beijing Training Project For The Leading Talents in S & T (NO.Z141107001514002).

## Conflict of interest

No conflict of interest.
